# Diabetic macular oedema and diode subthreshold micropulse laser (DIAMONDS): study protocol for a randomised controlled trial

**DOI:** 10.1186/s13063-019-3199-5

**Published:** 2019-02-12

**Authors:** Noemi Lois, Evie Gardner, Norman Waugh, Augusto Azuara-Blanco, Hema Mistry, Danny McAuley, Nachiketa Acharya, Tariq M. Aslam, Clare Bailey, Victor Chong, Louise Downey, Haralabos Eleftheriadis, Samia Fatum, Sheena George, Faruque Ghanchi, Markus Groppe, Robin Hamilton, Geeta Menon, Ahmed Saad, Sobha Sivaprasad, Marianne Shiew, David H. Steel, James Stephen Talks, Catherine Adams, Christina Campbell, Matthew Mills, Mike Clarke, On behalf of the DIAMONDS Study Group, Noemi Lois, Evie Gardner, Norman Waugh, Augusto Azuara-Blanco, Hema Mistry, Danny McAuley, Mike Clarke, Tariq M. Aslam, Clare Bailey, Tomas Burke, Victor Chong, Sobha Sivaprasad, David H. Steel, James Stephen Talks, Catherine Adams, Christina Campbell, Matthew Mills, Paul Doherty, Aby Joseph, Nachiketa Acharya, Seema Arora, Harbhajan Kaur Arora, Mandeep S. Bindra, Farahat Butt, Manju Chandran, Richard Cheong-Leen, Mark Costen, Bhatia Devangna, Louise Downey, Stefanos Efraimidis, Haralabos Eleftheriadis, Abdallah Ellaban, Samia Fatum, Mohamed Galal, Sheena George, Faruque Ghanchi, Kala Gopalakrishnan, Markus Groppe, Robin Hamilton, Maged S. Habib, Christine Kiire, Arun Kikkeri, Zeid Madanat, Krishnappa Madhusudhana, Sely Mathew, Ahmed Saad, Serena Salvatore, Peter Scanlon, Muhammad Shaikh, Marianne Shiew

**Affiliations:** 1 From The Wellcome-Wolfson Institute for Experimental Medicine, Queen’s University Belfast, 97 Lisburn Road, Belfast, BT9 7BL UK; 2 The Northern Ireland Clinical Trials Unit (NICTU), Belfast, UK; 3 The Division of Health Sciences, University of Warwick, Warwick, UK; 4 the Centre for Public Health, Queens University, Belfast, UK; 5 The Regional Intensive Care Unit, Royal Victoria Hospital, Belfast, UK; 6 Sheffield Teaching Hospitals NHS Foundation Trust, Sheffield, UK; 7 The Manchester Academic Health Science Centre, Manchester Royal Eye Hospital and Division of Pharmacy and Optometry, School of Health Sciences, Faculty of Biology, Medicine and Health, University of Manchester, Manchester, UK; 8 Bristol Eye Hospital, Bristol, UK; 9 Royal Free Hospital NHS Foundation Trust, London, UK; 10 Hull and East Yorkshire Hospital, Hull and East Yorkshire NHS Trust, Hull, UK; 11 Kings College Hospital NHS Foundation Trust, London, UK; 12 John Radcliffe Hospital, Oxford University Hospitals NHS Foundation Trust, Oxford, UK; 13 Hillingdon Hospitals NHS Foundation Trust, London, UK; 14 Bradford Teaching Hospitals NHS Trust, Bradford, UK; 15 Stoke Mandeville Hospital, Buckinghamshire NHS Trust, Aylesbury, UK; 16 Moorfields Eye Hospital NHS Foundation Trust, London, UK; 17 Frimley Park Hospital NHS Foundation Trust, Camberly, UK; 18 James Cook University Hospital, South Tees Hospitals NHS Foundation Trust, Middlesbrough, UK; 19 Zagazig University, Zagazig, Egypt; 20 NIHR Moorfields Biomedical Research Centre, London, UK; 21 Hinchingbrooke Hospital North West Anglia NHS Trust, Hinchingbrooke, UK; 22 Sunderland Eye Infirmary, Sunderland and Institute of Genetic Medicine, Newcastle University, Newcastle Upon Tyne, UK; 23 Newcastle Eye Centre and Newcastle upon Tyne Hospitals NHS Foundation Trust, Newcastle, UK

**Keywords:** Diabetes, Oedema, Edema, DMO, DME, Laser, Anti-VEGFs, Micropulse, RCT, Cost-effectiveness

## Abstract

**Background:**

In the UK, macular laser is the treatment of choice for people with diabetic macular oedema with central retinal subfield thickness (CST) < 400 μm, as per National Institute for Health and Care Excellence guidelines. It remains unclear whether subthreshold micropulse laser is superior and should replace standard threshold laser for the treatment of eligible patients.

**Methods:**

DIAMONDS is a pragmatic, multicentre, allocation-concealed, randomised, equivalence, double-masked clinical trial that aims to determine the clinical effectiveness and cost-effectiveness of subthreshold micropulse laser compared with standard threshold laser, for the treatment of diabetic macular oedema with CST < 400 μm. The primary outcome is the mean change in best-corrected visual acuity in the study eye from baseline to month 24 post treatment. Secondary outcomes (at 24 months) include change in binocular best corrected visual acuity; CST; mean deviation of the Humphrey 10–2 visual field; change in percentage of people meeting driving standards; European Quality of Life-5 Dimensions, National Eye Institute Visual Functioning Questionnaire-25 and VisQoL scores; incremental cost per quality-adjusted life year gained; side effects; number of laser treatments and use of additional therapies.

The primary statistical analysis will be per protocol rather than intention-to-treat analysis because the latter increases type I error in non-inferiority or equivalence trials. The difference between lasers for change in best-corrected visual acuity (using 95% CI) will be compared to the permitted maximum difference of five Early Treatment Diabetic Retinopathy Study (ETDRS) letters. Linear and logistic regression models will be used to compare outcomes between treatment groups. A Markov-model-based cost-utility analysis will extend beyond the trial period to estimate longer-term cost-effectiveness.

**Discussion:**

This trial will determine the clinical effectiveness and cost-effectiveness of subthreshold micropulse laser, when compared with standard threshold laser, for the treatment of diabetic macular oedema, the main cause of sight loss in people with diabetes mellitus.

**Trial registration:**

International Standard Randomised Controlled Trials, ISRCTN17742985. Registered on 19 May 2017 (retrospectively registered).

**Electronic supplementary material:**

The online version of this article (10.1186/s13063-019-3199-5) contains supplementary material, which is available to authorized users.

## Background

Diabetic macular oedema (DMO) is a leading cause of blindness in people with diabetes mellitus. It represents the accumulation of fluid at the macula, the area of the retina responsible for central vision. As fluid accumulates, visual loss ensues. Macular laser was, until recently, the treatment of choice for people with DMO. The Early Treatment Diabetic Retinopathy Study (ETDRS) demonstrated the beneficial effects of laser in 1985 [1]. The ETDRS showed that macular laser reduced the risk of visual loss (loss of ≥ 3 lines) in patients with clinically significant diabetic macular oedema (CSMO) by 50% at 3 years [1]. Only a few patients (3%) experienced visual acuity improvement of ≥ 15 letters but 85% of the eyes studied had vision of ≥ 20/40 at baseline, which could have accounted for the limited visual improvement observed [1]. More recent trials have shown higher rates of visual improvement (≥ 10 letters in 32% of patients at 2 years and in 44% at 3 years) [2, 3] suggesting that macular laser can indeed improve vision. 

Anti-vascular endothelial growth factor (anti-VEGF) therapies may be an alternative to laser treatment. The National Institute for Health and Care Excellence (NICE) in the UK reviewed ranibizumab (Lucentis®) and aflibercept (Eylea®) for the treatment of DMO, in 2013 (TA274) and 2015 (TA346), respectively [4, 5].NICE concluded these therapies were superior to macular laser for people with central retinal thickness ≥ 400 μm (CRT), as determined by spectral domain optical coherence tomography (SD-OCT), and, thus, recommended these drugs for this group of patients. However, for people with CRT < 400 μm, the cost-effectiveness evaluation showed laser treatment dominated and, hence, it remains the treatment of choice in the latter group. The NICE appraisal of ranibizumab in DMO used data from the RESTORE study, in which subgroups were pre-specified by retinal thickness < 300 μm, 300–400 and > 400. There was no significant difference between ranibizumab and laser in the < 300 μm group, but ranibizumab was much more effective than laser in the > 400 group, in which there was no improvement in best corrected visual acuity (BCVA) with laser. In the intermediate 300–400 μm group, ranibizumab was somewhat more effective than laser, with gains of ~ 8 and 4 letters in the ranibizumab and laser groups, respectively; the difference was statistically significant but of no clinical relevance. The much higher cost of ranibizumab meant that the cost per quality-adjusted life year (QALY) was very high and NICE did not consider ranibizumab to be cost-effective in the < 400 groups. Interestingly, in recent clinical trials comparing ranibizumab and aflibercept with laser, the mean CRT on average was > 400 μm (405 μ DRCR.net protocol I; > 460 μ RISE and RIDE; 412–426 μ RESTORE; > 479 μ VISTA and VIVID) [6–9]. Macular laser is also used in patients with DMO who do not fully respond to anti-VEGF therapy; in randomised trials, 41–64% of the eyes studied receiving anti-VEGF therapy required macular laser by 2 years after start of treatment [10].

Standard threshold macular laser is performed using a continuous wave laser that produces a visible burn in the retina. The laser energy is predominantly absorbed by one of the layers of the retina, the retinal pigment epithelium (RPE), and converted into heat. Although the mechanisms of action of conventional threshold laser are not completely understood, it is believed that it acts upon still-viable RPE cells around the site of the burn. As heat spreads by conduction, there is a potential for damage to the retinal layers overlying the RPE, including the photoreceptors (light-sensitive cells). Standard laser requires considerable expertise by the clinician who needs to identify, by slit-lamp biomicroscopy and with the help of SD-OCT and fundus fluorescein angiography (FFA), areas involved at which the laser should be aimed. Side effects of standard threshold macular laser are rare but include paracentral scotomas (areas around the central vision in which patients do not see, which may affect reading and driving), reduced colour vision and epiretinal membrane/subretinal fibrosis. If the centre of the macula is accidentally treated with laser (foveal burn), this will likely result in marked visual loss. If strong laser is applied close to the centre of the macula, subsequent atrophy (which could expand over the years to the centre) could similarly lead to loss of central vision.

Unlike in standard threshold laser, in subthreshold micropulse laser a series of repetitive very short laser pulses are applied. Each pulse is separated by a long off-time, which reduces the increased temperature in the tissue that follows conventional laser; a sublethal effect on the RPE is achieved with preservation of the overlying neurosensory retina, including the photoreceptors. Small case series and randomised trials including small numbers of patients have shown that subthreshold micropulse laser may be comparable or more efficacious than standard laser, with reduced side effects. For example, Lavinsky and collaborators, showed the superiority of high-density subthreshold micropulse laser in visual acuity improvement and reduced CRT at 12 months in a trial of participants randomised to this treatment (*n* = 42), to standard threshold laser (n = 42) or to low-density subthreshold micropulse laser (*n* = 39) [11]. In another trial including 50 patients with DMO, Vujosevic and colleagues found no differences in vision or CRT between patients randomised to standard threshold laser or subthreshold micropulse laser, but there was statistically significantly increased retinal sensitivity on microperimetry following the latter, with no laser scars in the retina at 12-month follow up [12]. Randomised trials by Kumar and associates, [13] Figueira and coworkers [14] and Laursen and collaborators [15] including 20, 53 and 16 patients, respectively, and with follow up of 18 weeks, 12 months and 5 months, respectively, found no differences in vison and CRT between the two types of laser. A recently published review [16] concluded that the available data suggests that subthreshold micropulse laser has similar or superior efficacy to standard threshold laser, with less or with no retinal damage. Subthreshold micropulse laser may allow also for more standardised delivery of treatment, given that it is applied to the entire macular area in a confluent manner, it reduces/minimises possible variability and is less dependent on the surgeon’s skills. Sight loss as a result of a foveal burn is obviated if subthreshold micropulse laser is used.

In summary, the published data suggest that subthreshold micropulse laser may be superior to conventional threshold laser, but stronger evidence is required to determine whether this is the case and whether this form of laser should be used instead of conventional threshold laser for the treatment of DMO.

## Subjects and methods

Ethical approval for this study was granted by the Office for Research Ethics Committees Northern Ireland (REC Reference 16/NI/0145).

### Study design and setting

DIAMONDS is a pragmatic, multicentre, allocation-concealed, randomised, equivalence, doubl*e*-masked clinical trial set within specialised Hospital Eye Services (HES) in the UK (see list of participating centres below). It aims to evaluate the clinical effectiveness and cost-effectiveness of subthreshold micropulse laser, when compared with standard threshold laser, for the treatment of patients with DMO with CST < 400 μm.

### Participants: eligibility criteria

Potential study participants will be identified through patient electronic databases at each participating centre, through referrals to HES or while in the clinic. Patients identified through electronic databases or referrals will be approached by phone or via an invitation letter. Verbal and written information about the study will be given to the participant and informed consent will be sought at their next clinical appointment; if provided, the patient will be recruited into the study. Patients identified while in clinic will be verbally informed about the study and they will receive a patient information leaflet. They will be given time to think about their participation and ask questions about the study. If they wish to go ahead and be enrolled on the same day, they will be recruited into the trial following informed consent. If someone would like more time to think about their potential participation in the study, a further visit will be organised for them. If, at this visit, they are willing to participate, they will be recruited.

Informed consent to participate in the study will be obtained by the ophthalmologist or a designee at each participating site. The treating ophthalmologist will obtain informed consent to carry out the laser procedure(s).

#### Inclusion criteria

Eligible participants will have DMO at the centre of the macula, as determined by slit-lamp biomicroscopy and SD-OCT, in one or both eyes with either:

1. CST of > 300 but < 400 microns as determined by SD-OCT due to DMO or
2. CST of < 300 microns provided that intraretinal and/or subretinal fluid is present in the central subfield (central 1 mm) related to DMO

They will also:

1. Have visual acuity of > 24 Early Treatment Diabetic Retinopathy Study (ETDRS) letters (Snellen equivalent > 20/320)
2. Be amenable to laser treatment, as judged by the treating ophthalmologist
3. Be over 18 years of age

#### Exclusion criteria

The participants’ eyes will not be eligible for the study if the macular oedema is due to causes other than DMO or if the eye is:

1. Ineligible for macular laser treatment, as judged by the treating ophthalmologist
2. Has DMO and CST of 400 μm or above
3. Has active proliferative diabetic retinopathy (PDR) requiring treatment
4. Has received intravitreal anti-VEGF therapy within the previous 2 months
5. Has received macular laser treatment within the previous 12 months
6. Has received intravitreal injection of steroids
7. Has had cataract surgery within the previous 6 weeks
8. Has had panretinal photocoagulation (PRP) within the previous 3 months

Otherwise eligible patients will not be included in the study if they:

1. Are on pioglitazone (as this drug could potentially be responsible for the presence of macular oedema) and the drug cannot be stopped 3 months prior to entering into the trial and for the duration of the study
2. Have chronic renal failure requiring dialysis or kidney transplant
3. Have any other condition that in the opinion of the investigator would preclude participation in the study (such as unstable medical status or severe disease that would make it difficult for the patient to be able to complete the study)
4. Have very poor glycemic control that required starting intensive therapy within the previous 3 months
5. Are using an investigational drug

If both eyes are eligible, both will receive the same type of laser but one will be designated the ‘study eye’. This will be the eye with best visual acuity at randomisation or, if vision is the same in both eyes, the eye with lesser CST.

If the fellow eye is not eligible, baseline data and information on whether participants develop DMO or PDR during the period of the study in the fellow eye and on treatments administered to it will be collected in the case report form (CRF) at months 12 and 24, to determine any possible effects of these events on outcomes.

### Outcome measures

#### Primary outcome

- Mean change in BCVA in the study eye at 24 months following treatment

#### Secondary outcomes

- Mean change in binocular best-corrected distance visual acuity (BCdVA) from baseline to month 24
- Mean change in CST in the study eye, as determined by SD-OCT, from baseline to month 24
- Mean change in the mean deviation (MD) of the Humphrey 10–2 visual field in the study eye from baseline to month 24
- Change in the percentage (%) of people meeting UK driving standards from baseline to month 24
- Mean change in EuroQoL(EQ-5D 5 L), the National Eye Institute Visual Function Questionnaire (NEI VFQ25) and VisQoL scores from baseline to month 24
- Incremental cost per QALY gained
- Side effects
- Number of laser treatments performed
- Use of additional treatments (other than laser)

Potential participants will be identified through referrals to participating HES or in ophthalmic clinics. Verbal and written information about the study will be provided. Informed consent will be obtained by the local principal investigator or designee at each site from those willing to participate. The schedule of visits and tests undertaken in DIAMONDS follows routine clinical practice; this should facilitate recruitment and retention of participants in the trial. In order to ensure adequate attendance of participants to follow-up visits, participants will be reminded by telephone, text or call the week prior to the study visit. This will be carried out by either research nurses or by administrative staff at each of the participating centres.

### Randomisation, interventions and study procedures

#### Randomisation

Participants will be randomised 1:1 using an automated randomisation system to receive either subthreshold micropulse laser (577 nm) or standard threshold laser, with the allocation concealed to the ophthalmologist randomising the patient until the patient has joined the trial. The local ophthalmologist will interact with this automated system to ensure masking of the outcome assessors to the allocation after randomisation. Although it is envisaged that most patients will receive laser at the baseline visit, laser can be performed within 2 weeks of that visit. If there is an interval between the baseline visit and the laser treatment, eligibility will be re-confirmed prior to undertaking the laser. Randomisation is advised to be conducted on the day of laser treatment.

The randomisation system will use a minimisation algorithm to ensure balanced allocation of patients across treatment groups for the following important prognostic factors: centre, distance BCdVA at presentation [≥ 69 ETDRS letters (Snellen equivalent ≥ 20/40; Logarithm of the Minimum Angle of Resolution (logMAR) ≥ 0.3); 24–68 ETDRS letters (Snellen equivalent ≤ 2 0/50; logMAR 0.4–1.2)], previous use of anti-VEGFs or macular laser in the study eye.

#### Intervention: laser treatment strategy and retreatments

Standard laser will be applied to areas of thickened retina, macular non-perfusion (away from and non-contiguous with the perifoveal capillaries) and leaking microaneurysms, in accordance with the Royal College of Ophthalmologist guidelines [17]. FFA and OCT will be used to identify areas of non-perfusion and leakage (FFA) and thickening (OCT) prior to treatment at the discretion of the treating ophthalmologist. Treatment will be applied to obtain a mild grey-white burn evident beneath leaking microaneurysms and in other areas of leakage/non perfusion not affecting the perifoveal capillaries based on FFA, if FFA has been obtained, and/or to cover areas of thickening if treatment is given based on OCT findings. Treatment will spare the central 500 μm and the area within 500 μm from the optic nerve head.

The majority of prospective, randomised controlled clinical trials on micropulse subthreshold laser therapy for the treatment of DMO have been performed with the 810 nm infrared diode laser [11, 12, 14, 15, 18]. Available data suggest similar efficacy and safety between 810 nm and 577 nm micropulse laser therapy for DMO [19, 20]. In DIAMONDS, micropulse laser therapy will be delivered with a 577 nm optically pumped diode laser (IQ 577™ laser system; IRIDEX Corporation). Subthreshold micropulse laser will be applied confluently to the macular area, using three 7 × 7 spot grids above and below the fovea (500 μm from its centre) and one 7 × 7 spot grid at each side (temporal and nasal) of the fovea (500 μm from its centre); treatment will also be applied to areas of thickening located outside this central area. First, a threshold will be set by titrating the power of the laser upwards, starting from 50 mW, in 10 mW increments, in an area where oedema is present, around > 2 DD (disc diameters) from foveal center (if possible), and until a barely visible tissue reaction is seen. If a reaction is evident with 50 mW, the power will not be increased. Then, the laser will be turned into the micropulse mode; on micropulse, the power of the laser will be set at × 4 the threshold identified (e.g. if a barely visible reaction is seen at 50 mW, then micropulse laser will be applied with 200 mW power).

Application of standard threshold and micropulse subthreshold laser will follow the DIAMONDS guidelines set for this purpose. All details of the laser procedure(s) will be recorded in an appropriately designed CRF including, among other details, the eye to be treated, the type of laser and laser parameters used, the name and grade of the physician conducting the treatment and the time spent applying the treatment.

Retreatments with laser can and should be undertaken, if necessary. All retreatments should use the same laser as determined by randomisation. The treatment of areas within 300–500 μm from the centre of the fovea is allowed when retreating. Details of retreatments should be documented in the case report form (CRF).

Rescue treatment (with anti-VEGFs or steroids as appropriate) will be allowed in both treatment groups if the CST increases to 400 μm or over at any point during the follow up or if a loss ≥ 10 ETDRS letters occurs related to DMO. Rescue treatments will be recorded (type and date) in the CRF.

#### Study procedures: patient evaluation

All patients will be evaluated during the study according to the schedule of assessments shown in Table 1. BCVA will be measured in both eyes using ETDRS visual acuity charts at 4 m at baseline and at months 4, 8, 12, 16, 20 and 24. BCVA will be obtained following refraction at baseline, 12 and 24 months by optometrists masked to treatment allocation. At all other visits, BCVA could be obtained by other masked staff using the most recently obtained refraction. Binocular BCVA will be obtained also to give indication of the person’s vision in real life, using both eyes; it will be obtained by masked optometrists using the ETDRS visual acuity charts at 4 m at baseline and 12 and 24 months. A refraction protocol (as set in the DIAMONDS Visual Acuity Guideline contained in the Trial Manual) will be followed by the DIAMONDS optometrists to obtain BCVA. ETDRS visual acuity scores will be recorded for study eyes and fellow eyes in the appropriate CRF at each study visit.

**Table 1:** Schedule of assessments and procedures

	Baseline^b^	Post randomisation (months)					
		4^b^	8^b^	12^b^	16^b^	20^b^	24^b^
Informed consent	✓						
Medical history	✓	✓	✓	✓	✓	✓	✓
HbA1c^a^	✓						
BCVA in study eye and fellow eye	✓	✓	✓	✓	✓	✓	✓
Binocular distance vision	✓			✓			✓
Humphrey 10–2 visual field in study eye	✓			✓			✓
Esterman binocular visual field	✓			✓			✓
SD-OCT	✓	✓	✓	✓	✓	✓	✓
NEI VFQ-25	✓			✓			✓
EQ-5D-5L	✓			✓			✓
VisQol	✓			✓			✓
Randomisation	✓						
Subthreshold micropulse laser/ standard threshold laser	✓	^$^	^$^	^$^	^$^	^$^	^$^
Adverse events	✓	✓	✓	✓	✓	✓	✓

*BCVA* best corrected visual acuity, *SD-OCT* spectral domain optical coherence tomography, *NEI-VFQ-25* National Eye Institute Visual Functioning Questionnaire-25, *EQ-5D-5 L* European Quality of Life-5 Dimensions, *$* retreatment is possible at follow-up visits if needed

^a^If glycosylated haemoglobin type A1C (HbA1c) has been tested in the past 3 months and its value is available, it can be recorded in the case report form. If no previous HBA1c test (within the previous 3 months from baseline), a blood sample should be drawn to measure it. HbA1c is obtained as a measure of glycaemic control

^b^Visits may take place within ± 14 days of the due date

The study eye or both eyes (if both are included) will undergo 10–2 Humphrey visual field testing by a visual field technician masked to the allocated treatment at baseline and at months 12 and 24. The Esterman binocular visual field (to determine patient’s ability to fulfil driving standards) will be obtained at the same time points. Visual fields eligible for analysis will have to achieve pre-defined reliability criteria (false positives < 15%). If the visual fields are not reliable they should be repeated. The mean deviation (MD) value for the 10–2 Humphrey visual fields and the number of points seen/missed in the Esterman binocular visual fields will be recorded in the CRF.

CST, as determined by using SD-OCT, will be obtained in both eyes at baseline and at months 4, 8, 12, 16, 20 and 24. SD-OCT will be obtained by technicians, photographers or nurses, as per standard clinical practice at each of the participating centres, masked to the treatment allocation. The measure of thickness at the central 1 mm (i.e. CST) will be recorded in the CRF and used for analysis. In addition, total and maximal macular volume, will be recorded in the CRF. Presence or absence of intraretinal or subretinal fluid will be determined in a masked fashion by masked readers at the Central Angiographic Resource Facility (CARF) at Queens University, Belfast at the 24-month follow-up visit. Images sent to CARF will be anonymised. The same SD-OCT machine should be used to obtain the above measurement for each patient at baseline and at each of the follow-up visits.

We will use two vision-related quality of life tools: the NEI VFQ-25 and the VisQol questionnaires. We will also use the generic preference-based health-related quality of life measure EQ-5D-5 L to generate utility data. All questionnaires will be self-completed by patients at baseline and at 12 and 24 months. The baseline questionnaires should be completed before the first session of laser treatment (subthreshold micropulse or threshold standard laser).

As stated previously, outcome assessors and also participants will be masked to the treatment allocation. Participants will be followed at 4-month intervals following laser for a total of seven visits, which is in accordance with routine standard clinical care. Additional visits (interim visits) may occur, if required.

In order to maximise retention in the study, DIAMONDS was designed as a pragmatic trial, with visits every 4 months, as stated previously, as in usual, routine care. In most visits, with the exception of those at baseline, 12 and 24 months, the tests will be the same as those done in routine practice (i.e. measure of visual acuity and SD-OCT scans). Furthermore, participants will be reminded of their clinical appointment by telephone, text or call a week before the study visit.

**Fig. 1:**
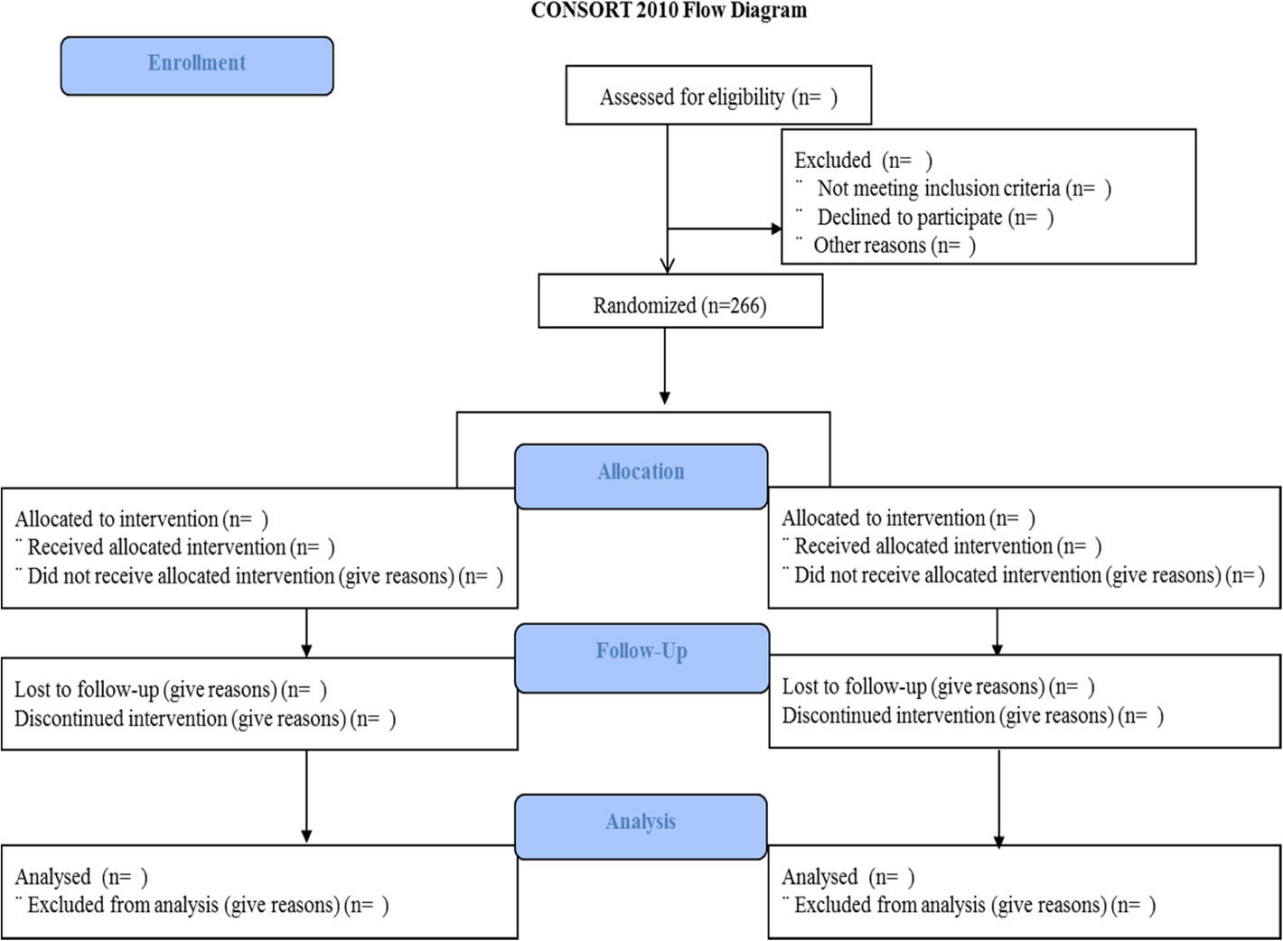
DIAMONDS Consolidated Standards of Reporting Trials (CONSORT) flow diagram

A CONSORT diagram will be presented for the study, as shown in Fig. 1.

### Data collection and quality checks

CRFs will be used to collect data for the trial. On-site monitoring visits during the trial will check the accuracy of entries in the CRF against the source documents, the adherence to the protocol, procedures and to the International Conference of Harmonisation Good Clinical Practice (ICH-GCP) guidelines and regulatory requirements. Monitoring visits will be undertaken by a monitor from the Northern Ireland Clinical Trials Unit (NICTU). To ensure accurate, complete and reliable data are collected, the Chief Investigator and the NICTU will provide training to site staff through investigator meetings and site initiation visits.

Data quality control checks will be carried out by a data manager following standard operating procedures set at the NICTU, to ensure accuracy, and data errors will be documented in quality control reports with corrective actions implemented. Data validation will be implemented and discrepancy reports will be generated following data entry to identify discrepancies such as out of range, inconsistencies or protocol deviations based on data validation checks programmed in the clinical trial database.

Data obtained in DIAMONDS will be made available to the scientific community with as few restrictions as possible; the DIAMONDS group, however, will retain exclusive use until the major outputs have been published. Anonymised data will be deposited in the DIAMONDS website.

### Sample size

DIAMONDS is powered to demonstrate non-inferiority of subthreshold micropulse laser with respect to the primary outcome (BCVA in the study eye at 24 months). The trial will have sufficient statistical power to determine superiority of one laser treatment over the other. Furthermore, it will also have sufficient statistical power to determine equivalence of the types of laser treatment, because it is possible that even if no differences in the primary outcome are observed between the two, there may be differences in important secondary outcomes (e.g. patient reported outcomes (PROS)).

Based on a mean (standard deviation; SD) of 0.08 (0.23) logMAR for BCVA change from baseline for the standard care laser [11] and a permitted maximum difference of 0.1 logMAR (5 ETDRS letters) between groups, we estimated that DIAMONDS will require 113 randomised participants per group, at 90% power and 0.05 level of significance. Allowing for up to 15% dropout rate during the 24 months of follow-up, as observed in other randomised trials on DMO with outcomes determined at that time point [7, 21] a total of 266 patients will be recruited.

A permitted maximal difference of 5 ETDRS letters between groups was chosen as the non-inferiority margin because a 5 ETDRS letter or less difference is not considered clinically relevant or meaningful to patients [4, 5].

If data are available for 113 patients per group, this will also be sufficient to detect a mean difference between lasers of 37.7 μm in CST (based on SD of 86.8 [12]) and of 6.55 in the NEI-VFQ (based on SD of 15.1 as per previous publication [22]). These are important secondary outcomes for DIAMONDS and such differences in CST and NEI-VFQ-25 scores have both been shown to be clinically relevant differences [23, 24].

### Data analysis plan

The primary statistical analysis will be per protocol rather than an intention-to-treat (ITT) analysis. ITT analysis, which is recommended for superiority trials, will be performed but per-protocol analysis is preferred for non-inferiority or equivalence trials [25] because ITT increases the type I error in such trials.

The difference between laser treatments for change in BCVA (using 95% CI) from baseline to month 24 (primary endpoint) will be compared to the permitted maximum difference of 5 ETDRS letters (0.1 logMAR). The subthreshold micropulse laser can be deemed to be non-inferior to the standard laser if the lower limit of the 95% confidence interval of the treatment difference lies above the non-inferiority margin. If the 95% confidence interval of the treatment difference lies wholly within both the upper and lower margins of the permitted maximum difference (+/− 5 ETDRS letters), then subthreshold micropulse laser can be deemed to be equivalent to the standard laser. Change in BCVA from baseline to month 24 will be compared between the two laser groups using an analysis of covariance (ANCOVA) model adjusted for baseline BCVA score, baseline CST and minimisation factors/covariates including centre, distance BCVA at presentation [≥ 69 ETDRS letters (Snellen equivalent ≥ 20/40; logMAR ≥ 0.3) or 24–68 ETDRS letters (Snellen equivalent ≤ 20/50; logMAR 0.4–1.2)] and previous use of anti-VEGFs or laser in the study eye. The primary analysis will be based on data from the study eye only. When performing a secondary analysis on the subset of subjects with both eyes treated, study eye will be included as a random effect within the mixed model. Statistical diagnostic methods will be used to check for violations of the model assumptions and data transformations or non-parametric equivalents such as the Mann-Whitney test may be performed as appropriate.

Statistical significance will be based on two-sided tests, with *P* < 0.05 taken as the criterion for statistical significance. The principal analysis will be based upon available case data with no imputation of missing values. Sensitivity will be analysed to assess the impact of missing data by imputing extreme values (lowest and highest). Additionally, the primary outcome will be analysed according to pre-specified subgroups (previous use of anti-VEGFs or macular laser in the study eye) by including the corresponding interaction term in the regression model using stricter criteria for statistical significance (*P* ≤ 0.01). Side effects of the treatment and use of additional treatments will be analysed using logistic regression models with adjustment for the minimisation covariates. Health-related and visual-related quality of life measures, secondary measures of visual function, anatomical outcomes and number of treatments required will be analysed using linear regression models adjusted for baseline BCVA score and minimisation variables. “Driving ability” (meeting standards for driving) will be analysed using a logistic regression model adjusted for baseline BCVA and the minimisation variables.

Using random allocation and a standard care arm, we aim to reduce regression to the mean (RTM) in the design stage and we will use ANCOVA as a secondary analysis to account for possible effects of RTM. Baseline characteristics, follow-up measurements and safety data will be described graphically and in tabular format using descriptive summary measures depending on the scale of measurement and distribution. A detailed statistical analysis plan (SAP) will be written by the trial statistician prior to the final analysis.

### Health economic analysis

An economic evaluation will be conducted alongside the trial with a 2-year time horizon from a National Health Service (NHS) and personal social services perspective to estimate the cost per QALY gained of subthreshold micropulse compared with standard threshold laser. This will take into account utility gains from preservation or improvement in vision, and disutilities from adverse effects, including any effects on anxiety. Resource use of the different laser treatments, including staff time, equipment required, overheads, consumables, any rescue treatments and any other visits or resources used due to DMO for each patient will be recorded on the CRFs. Unit cost information obtained from published sources and centres participating in the trial will be attached to each resource item in order to calculate a cost for each trial patient. QALYs will be calculated as the area under the baseline-adjusted utility curve, and will be calculated using linear interpolation between baseline and follow-up utility scores.

A Markov model will be used to extrapolate beyond the trial, with costs and benefits discounted at 3.5%. The model will be populated by data from the trial and supplemented by estimates from published literature and expert opinion. Results will be expressed as cost per QALY gained. We will use sensitivity analyses to assess the robustness of the results, with probabilistic sensitivity analyses to explore uncertainty in model parameters and allow the presentation of cost-effectiveness acceptability curves.

### Patient and public involvement (PPI) in DIAMONDS

A PPI group (DIAMONDS PPI) was created very early on, at the stage of trial conception. The PPI provided input on trial design and outcome measures important to patients with DMO.

## Discussion

Progress made so far (as per September 28, 2018): recruitment has started at all participating sites (*n* = 16). Eleven sites were originally opened to recruitment; five others were added at a later date to ensure full recruitment would be achieved in the recruitment period of the trial. A total of 233 participants (out of the 266 required) have been recruited into the trial.

## Trial status

The current protocol is Protocol v4.0 22 November 2017. Recruitment started on 18 January 2017. The anticipated date of recruitment completion is 18 December 2018.

This protocol was prepared following standard protocol items: recommendation for interventional trials (Additional file 1) guidelines [26]; items listed in the World Health organisation Trial Registration Data Set have been specified throughout this protocol. A SPIRIT checklist has been provided with this manuscript.

## Additional files

Additional file 1: Reporting checklist for protocol of a clinical trial. (DOCX 24 kb)

Additional file 2: Roles and responsibilities of the Trial Steering Committee (TSC) and Data monitoring and Ethics Committee (DMEC). (PDF 434 kb)

## Abbreviations

ANCOVA: Analysis of covariance
Anti-VEGF: Anti-vascular endothelial growth factor
AR: Adverse reaction
BCdVA: Best corrected distance visual acuity
BCVA: Best corrected visual acuity
CARF: Central Angiographic Resource Facility
CRF: Case report form
CST: Central retinal subfield thickness
DMEC: Data Monitoring and Ethics Committee
DMO: Diabetic macular oedema
DSML: Diode subthreshold micropulse laser
EQ-5D-5 L: European Quality of Life 5 Dimensions
ETDRS: Early treatment diabetic retinopathy study
FFA: Fundus fluorescein angiography
GCP: Good Clinical Practice
HbA1c: Glycosylated hemoglobin type A1C
HES: Hospital Eye Services
HTA: Health Technology Assessment
ICH: International Conference on Harmonisation
ISRCTN: International Standard Randomised Controlled Trial Number
ITT: Intention to treat
LogMAR: Logarithm of the Minimum Angle of Resolution
MD: Mean deviation
ND: YAG: Neodymium-doped yttrium aluminium garnet
NEI-VFQ-25: National Eye Institute Visual Functioning Questionnaire-25
NHS: National Health Service
NICE: National Institute of Health and Care Excellence
NICTU: Northern Ireland Clinical Trials Unit
NIHR: National Institute of Health Research
OCT: Optical coherence tomography
PDR: Proliferative diabetic retinopathy
QALY: Quality-adjusted life year
RCT: Randomised controlled trial
REC: Research Ethics Committee
RPE: Retinal pigment epithelium
SAE: Serious adverse event
SAP: Statistical analysis plan
SD-OCT: Spectral domain optical coherence tomography
TSC: Trial Steering Committee
