# Supplementary material for: Diabetic macular oedema and diode subthreshold micropulse laser (DIAMONDS): study protocol for a randomised controlled trial
Source: Trials. 2019 Feb 12;20:122. doi: 10.1186/s13063-019-3199-5 (PMC6373040; doi:10.1186/s13063-019-3199-5)
Supplement: Supplementary file 2 — Roles and responsibilities of the Trial Steering Committee (TSC) and Data monitoring and Ethics Committee (DMEC). (PDF 434 kb) [file 13063_2019_3199_MOESM2_ESM.pdf]

RESEARCH GOVERNANCE GUIDELINES

Please contact your nominees before you give us their names, to ascertain their availability and willingness to be appointed.

TRIAL STEERING COMMITTEE (TSC) or STUDY STEERING COMMITTEE (SSC)

All primary research projects are required to establish a TSC/SSC. The following lists provide guidance on the role, constitution, composition, meeting requirements and primary reporting line for a TSC/SSC. This information can be used as a template terms of reference.

The role of the TSC/SSC

The role of the TSC/SSC is to provide overall supervision for a project on behalf of the Project Sponsor and Project Funder and to ensure that the project is conducted to the rigorous standards set out in the Department of Health's Research Governance Framework for Health and Social Care and the Guidelines for Good Clinical Practice. It should be noted that the day-to-day management of the project is the responsibility of the Chief Investigator, and as such the Chief Investigator may wish to set up a separate Project Management Group (PMG) to assist with this function.

The main features of the TSC/SSC are as follows:

- To provide advice, through its Chair, to the Trial/Project Funder, the Trial/Project Sponsor, the Chief Investigator, the Host Institution and the Contractor on all appropriate aspects of the project
- To concentrate on progress of the trial/project, adherence to the protocol, patient safety (where appropriate) and the consideration of new information of relevance to the research question
- The rights, safety and well-being of the participants are the most important considerations and should prevail over the interests of science and society
- To ensure appropriate ethical and other approvals are obtained in line with the project plan
- To agree proposals for substantial protocol amendments and provide advice to the sponsor and funder regarding approvals of such amendments
- To provide advice to the investigators on all aspects of the trial/project.

Constitution of a TSC/SSC

- The relevant NIHR Programme Director will review the nominees and appoint the Chair and members
- All TSC/SSC meetings are to have a minimum of 75% majority of independent* members

- The minimum quoracy for a meeting to conduct business is 67% of appointed members
- Only appointed members will be entitled to vote and the Chair will have a casting vote
- The Chair and members to sign and maintain a log of potential conflicts and/or interests
- Attendance at TSC/SSC meetings by non-members is at the discretion of the Chair
- The primary TSC/SSC reporting line is via the Chair to the relevant NIHR Programme Director; however communication is likely to be between the Chair and the NIHR Research Manager who has day to day responsibility for the project.

** Independence is defined as follows:*

- *Not part of the same institution as any of the applicants or members of the project team.*
- *Not part of the same institution that is acting as a recruitment or investigative centre, including Patient Identification Centres (PIC), identifying and referring patients to a recruitment or investigative centre.*
  (In both cases above 'not part of the same institution' means holding neither a substantive or honorary contract with said institution).
- *Not related to any of the applicants or project team members.*
- *For the Chair only; not an applicant on a rival proposal.*
- *It is recognised that independence status may change during the duration of the trial.*

Composition of the TSC/SSC

- An Independent* Chair (UK based and/or holding a substantive UK based appointment)
- Independent* statistician, health economist and clinician(s) and any others with expertise relevant to the project
- At least one individual who is able to contribute a patient and/or wider public perspective
- Ideally, the TSC/SSC should invite observers, including a representative of the sponsor and a representative from the research network to meetings
- An indication of any proposed overseas members should have been given at the full application stage and feedback on such proposals supplied following the Funding Board's consideration of the application.

TSC/SSC meetings

- Although there may be periods when more frequent meetings are necessary, the TSC/SSC should meet at least annually
- Where a DM(E)C is required, TSC/SSC meetings should be scheduled to follow shortly after DM(E)C meetings so that reports from that group can be considered if appropriate
- Minutes of meetings should be sent to all members, the sponsor, and the funder and be retained in the study master file.

The responsibility for calling and organising TSC/SSC meetings lies with the Chief Investigator, in association with the Chair.

There may be occasions when the Project Sponsor or the Project Funder will wish to organise and administer these meetings for particular projects. This is unlikely, but the

NIHR reserves the right to attend any meeting therefore should be included in relevant invitations and also reserves the right to convene a meeting of the TSC/SSC in exceptional circumstances.

The Role of the Chair of TSC/SSC

The Chair of the TSC/SSC is directly answerable to the relevant NIHR programme, as funder. The Chair's responsibilities include:

- Liaising with the Chief Investigator to arrange a meeting to finalise the protocol and to set up a schedule of meetings to align with the project plan
- Establishing clear reporting lines to the Funder, Sponsor, etc.
- Being familiar with relevant guidance documents and with the role of the DM(E)C if appropriate
- Providing an independent*, experienced opinion if conflicts arise between the needs of the research team, the funder, the sponsor, the participating organisations and/or any other agencies
- Leading the TSC/SSC to provide regular, impartial oversight of the study, especially to identify and pre-empt problems
- Ensuring that changes to the protocol are debated and endorsed by the TSC/SSC; letters of endorsement should be made available to the project team when requesting approval from the funder and sponsor for matters such as changes to protocol
- Being available to provide independent* advice as required, not just when TSC/SSC meetings are scheduled
- Commenting on any extension requests and, where appropriate, providing a letter to the funder commenting on whether the extension request is supported or otherwise by the independent* members of the TSC
- Commenting in detail (when appropriate) regarding the continuation, extension or termination of the project.

NB: The TSC/SSC Chair does not need to be a content expert him/herself but needs to ensure that sufficient content expertise is available for the group to perform its oversight function effectively.

DATA MONITORING (AND ETHICS) COMMITTEE (DM(E)C)

The formation of a Data Monitoring and Ethics Committee (DM(E)C) is needed when there are particular risk factors involved in a study.

Most primary research projects are required to establish a DM(E)C. The following lists provide guidance on the role, constitution, composition and meeting requirements for a DM(E)C. This information can be used as a template terms of reference.

The role of the DM(E)C

The DM(E)Cs main role is as follows:

- It is the only body involved in a trial that has access to the unblinded comparative data
- The role of its members is to monitor these data and make recommendations to the TSC/SSC on whether there are any ethical or safety reasons why the trial should not continue
- The safety, rights and well-being of the trial participants are paramount
- The DM(E)C considers the need for any interim analysis advising the TSC/SSC regarding the release of data and/or information
- The DM(E)C may be asked by the TSC/SSC, Project Sponsor or Project Funder to consider data emerging from other related studies
- There are also rare occasions when the DM(E)C chair might be asked by the Project Funder to provide a confidential interim or futility analysis if serious concerns are raised about the viability of the study or if the research team are requesting significant extensions.

Constitution of a DM(E)C

- The relevant NIHR Programme Director will review the nominees and appoint the Chair and members
- Only appointed members will be entitled to vote and the Chair will have a casting vote
- The minimum quoracy for a meeting to conduct business is 67% of appointed members
- The Chair and members to sign and maintain a log of potential conflicts and/or interests
- Attendance at DM(E)C meetings by non-members is at the discretion of the Chair
- The primary DM(E)C reporting line is via the Chair to the TSC/SSC.

Composition of a DM(E)C

- All DM(E)C members are to be independent* (with at least one member being UK based and/or holding a substantive UK based appointment)
- Membership of the DM(E)C should be small (3- 4 members) and comprise experts in the field, e.g. a clinician with experience in the relevant area and expert trial statistician.

DM(E)C Meetings

- Responsibility for calling and organising DM(E)C meetings lies with the Chief Investigator, in association with the Chair of the DM(E)C. The project team should

provide the DM(E)C with a comprehensive report, the content of which should be agreed in advance by the Chair of the DM(E)C

- The DM(E)C should meet at least annually, or more often as appropriate, and meetings should be timed so that reports can be fed into the TSC/SSC
- Minutes of meeting should be sent to all members, the sponsor, the funder, the TSC/SSC and the study master file. It should be noted that the minutes may have ‘in camera’ items redacted from some copies.

**Independence is defined as follows:*

- *Not part of the same institution as any of the applicants or members of the project team.*
- *Not part of the same institution that is acting as a recruitment or investigative centre, including Patient Identification Centres (PIC) identifying and referring patients to a recruitment or investigative centre.*
  (In both cases above ‘not part of the same institution’ means holding neither a substantive or honorary contract with said institution).
- *Not related to any of the applicants or project team members.*
- *For the Chair only; not an applicant on a rival proposal.*
- *It is recognised that independence status may change during the duration of the trial.*
